# A functional interplay between intein and extein sequences in protein splicing compensates for the essential block B histidine†
†Electronic supplementary information (ESI) available. See DOI: 10.1039/c8sc01074a


**DOI:** 10.1039/c8sc01074a

**Published:** 2018-10-03

**Authors:** Kristina Friedel, Monika A. Popp, Julian C. J. Matern, Emerich M. Gazdag, Ilka V. Thiel, Gerrit Volkmann, Wulf Blankenfeldt, Henning D. Mootz

**Affiliations:** a Institute of Biochemistry , University of Muenster , Wilhelm-Klemm-Str. 2 , 48149 Münster , Germany . Email: Henning.Mootz@uni-muenster.de; b Structure and Function of Proteins , Helmholtz Centre for Infection Research , Inhoffenstraße 7 , 38124 , Braunschweig , Germany; c Institute for Biochemistry, Biotechnology and Bioinformatics , Technische Universität Braunschweig , Spielmannstraße 7 , 38106 Braunschweig , Germany

## Abstract

Inteins remove themselves from a precursor protein by protein splicing. Due to the concomitant structural changes of the host protein, this self-processing reaction has enabled many applications in protein biotechnology and chemical biology. We show that the evolved M86 mutant of the *Ssp* DnaB intein displays a significantly improved tolerance towards non-native amino acids at the N-terminally flanking (–1) extein position compared to the parent intein, in the form of both an artificially *trans*-splicing split intein and the *cis*-splicing mini-intein. Surprisingly, side chains with increased steric bulk compared to the native Gly(–1) residue, including d-amino acids, were found to compensate for the essential block B histidine in His73Ala mutants in the initial N–S acyl shift of the protein splicing pathway. In the case of the M86 intein, large (–1) side chains can even rescue protein splicing activity as a whole. With the comparison of three crystal structures, namely of the M86 intein as well as of its Gly(–1)Phe and Gly(–1)Phe/His73Ala mutants, our data supports a model in which the intein's active site can exert a strain by varying mechanisms on the different angles of the scissile bond at the extein–intein junction to effect a ground-state destabilization. The compensatory mechanism of the block B histidine is the first example for the direct functional role of an extein residue in protein splicing. It sheds new light on the extein–intein interplay and on possible consequences of their co-evolution as well as on the laboratory engineering of improved inteins.

## Abstract 2

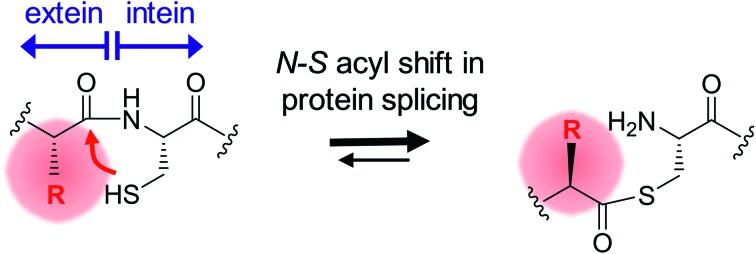
Steric bulk can compensate for a catalytically critical histidine in an intein's active site and promote the N–S acyl shift.

## Introduction

**Fig. 1:**
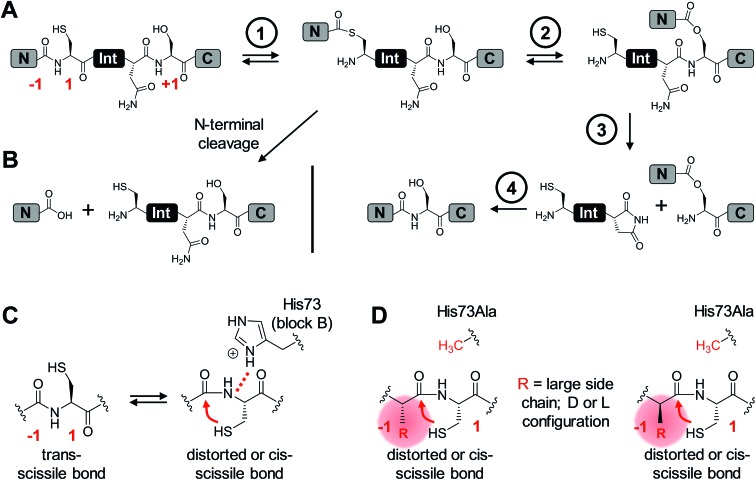
Mechanisms of protein splicing and the N–S acyl shift. (A) Mechanism of the protein splicing pathway of class 1 inteins. Shown here are Cys and Ser residues at the 1 and +1 positions, respectively, as present in the *Ssp* DnaB intein. Step 1: N–S acyl shift to the linear thioester intermediate; step 2: *trans*-esterification to the branched intermediate; step 3: asparagine cyclization to effect succinimide formation and cleavage of the C-terminal scissile bond; step 4: spontaneous O–N shift to the final splice product (uncatalyzed). (B) Side-reaction of N-terminal cleavage through nucleophilic attack of the linear thioester. This step is enforced when the C-terminal splice junction is mutated. (C) Mechanistic contribution of the essential block B histidine in the N–S acyl shift. (D) New artificial mechanisms to compensate for a lacking block B histidine as proposed in this work. Red numbers indicate the nomenclature in residue numbering at the extein–intein junctions.

Inteins are protein elements that remove themselves from their precursor proteins in an autocatalytic pathway called protein splicing.^1^–^3^ The mature host protein is produced by the ligation of the flanking sequences, referred to as N- and C-terminal exteins, with a native peptide bond. The posttranslational alteration of the peptide backbone and hence of the protein's structure has enabled a plethora of applications, many of which involve mutated or otherwise engineered inteins to control the protein splicing pathway or induce off-pathway cleavage reactions. Protein splicing is a multi-step process of intramolecular rearrangements that keeps the intein and extein segments covalently attached until the exteins are connected (Fig. 1A).^3^–^5^ In the first step of the canonical class 1 inteins,^6^ the scissile peptide bond upstream of the intein rearranges in an N–S (or N–O) acyl shift involving the cysteine (or serine) side chain as the first amino acid of the intein (residue 1) and the upstream flanking amino acid of the N-extein (residue (–1)). The resulting linear thioester (or oxoester) is then transesterified onto the sulfhydryl (or hydroxyl) side chain of a cysteine (or serine/threonine) as the first amino acid downstream of the intein, the (+1) residue, to give a branched intermediate. In the third step catalyzed by the intein, the asparagine (or glutamine) side chain preceding the C-terminal scissile bond attacks the downstream intein–extein junction to effect its cleavage with concomitant cyclization of the side chain. Finally, the thioester (or oxoester) bond connecting the N- and C-terminal exteins rearranges in an uncatalyzed S–N (or O–N) acyl shift to give a stable peptide bond in the mature splice product (Fig. 1A).

Inteins can be referred to as single-turnover enzymes because they employ typical enzymatic strategies to bring about the reactions. However, they do catalyze the reaction only once. Mutational studies on several inteins have led to the idea that inteins use varying strategies to catalyze the individual steps. In particular, conserved residues from conserved block motifs may contribute to a different extent when comparing different inteins. For example, in the case of the initial reaction, the N–S (or N–O) acyl shift, a histidine from the block B motif, also referred to as the N3 motif,^7^ is the single most highly conserved amino acid in inteins. It is believed to polarize the N-terminal scissile bond and thereby effect a ground-state destabilization (Fig. 1C),^8^–^11^ although other studies have suggested roles in deprotonation of the Cys1 side chain or the stabilization of the oxyanion of the tetrahedral intermediate.^12^ The block B histidine is essential in many inteins including the *Ssp* DnaB intein.^10^,^13^,^14^ However, in others, such as the *Pab* PolII intein, it is only important, *i.e.*, its mutation lowers but does not fully impair the efficiency of protein splicing.^15^ There is even a very small subset of inteins that lack the histidine side chain. In this case another residue, which is not important in inteins containing the block B histidine, acts in a compensatory mechanism, presumably by stabilizing the tetrahedral intermediate.^16^ Such a flexible catalysis might have been allowed during evolution because no repeated catalytic turnover is required and the selective pressure on a highly optimized catalytic strategy likely was limited.^16^

The initial N–S acyl shift in protein splicing is also of great interest in the field of chemical ligation by native chemical ligation or expressed protein ligation because a peptide or protein thioester is required here and interception of the protein splicing pathway is the method of choice for making C-terminal thioesters of recombinant proteins.^17^,^18^ Chemical strategies to facilitate acyl shift reactions for the preparation of thioester peptides include the use of an *N*-alkylated cysteine.^19^–^22^ An *N*-methyl cysteine in the active site of the *Ssp* DnaB intein was found to functionally compensate a block B histidine mutant.^10^

In this work, we have studied the extein sequence dependence of the *Ssp* DnaB intein^23^ and its evolved M86 mutant,^24^ both in *trans*- and in *cis*-splicing forms.^25^,^26^ By serendipity, we discovered a compensatory mechanism for the loss of the block B histidine that is dependent on the size of the upstream flanking (–1) residue. Crystal structures of an M86 precursor intein as well as of two extein mutants with and without the block B histidine help explain the compensatory mechanism by implying steric effects to distort the N-terminal splice junction.

## Results and discussion

### The M86 mutant intein displays increased splicing promiscuity with regard to the upstream flanking amino acid

**Fig. 2:**
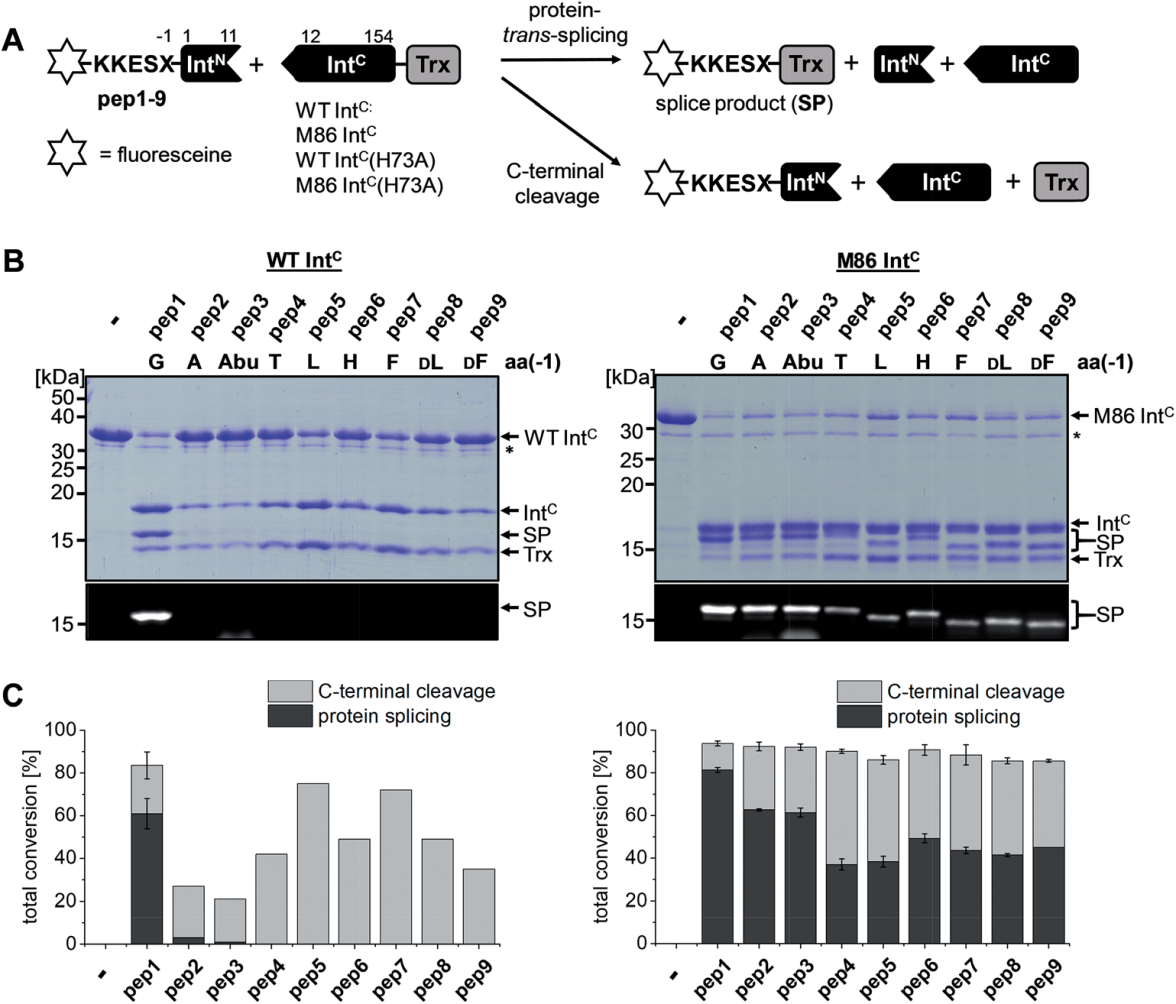
Extein dependence at the (–1) position of semisynthetic protein *trans*-splicing. (A) Scheme of the reactions involving the WT *Ssp* DnaB and M86 inteins. (B) Analysis of reactions on SDS-PAGE gels using Coomassie-staining (top) or UV illumination (bottom panel). Int^C^ proteins (20 μM) were incubated with the indicated peptides (60 μM) for 24 h and then quenched by SDS loading buffer and boiling. The variation of a single residue had a surprisingly large impact on the migration behavior of some of the splice products (SP). The weak bands visible for pep3 in the lower panels seem to result from a slight impurity of this peptide and were also observed in the absence of a protein partner (data not shown). (C) Yields of protein *trans*-splicing and C-terminal cleavage reactions determined by densitometric analysis of the Coomassie-stained gels shown in B. Error bars indicate standard deviations. Asterisks denote a protein contamination.

The M86 mutant was previously selected by sequential directed evolution from the parent *Ssp* DnaB intein^23^ as a more general protein splicing catalyst with regard to the flanking amino acids, a highly desirable trait when choosing a ligation site.^24^ Indeed, several studies indicated its increased sequence promiscuity, both in the form of the original mini-intein as well as an engineered split intein. In the latter case, an N-terminal fragment (Int^N^) of 11 amino acids and a C-terminal fragment (Int^C^) of 143 amino acids were generated.^24^,^27^–^29^ However, a systematic analysis of individual substitutions of the extein amino acids immediately flanking the intein has not been reported yet. To this end, we decided to study the tolerance towards the (–1) residue at the position upstream of the N-terminal scissile bond. We compared the parent *Ssp* DnaB intein and the M86 mutant in the context of the semi-synthetic split inteins. A set of synthetic Ex^N^–Int^N^ peptides pep1 to pep9 was synthesized with various residues of increasing steric bulk at the (–1) extein position, ranging from Gly as observed in the wild-type intein to large side chains and amino acids in the d-configuration (Table 1). 5,6-Carboxyfluorescein (Fl–) was included in the short extein^N^ sequence to facilitate analysis of the splicing and cleavage reactions. Note that all 8 mutations of the M86 intein are located in the Int^C^ fragment. Whereas the parent wild-type intein (WT Int^C^) with thioredoxin (Trx) as the model extein^C^ sequence was able to efficiently splice only with the native Gly(–1) residue, the corresponding M86 construct (M86 Int^C^) exhibited robust activity in all cases (Fig. 2 and Table 1). Table S1 and Fig. S1A† show the yields and the kinetic constants for these *trans*-splicing reactions. Additional fractions of the Int^C^ partner proteins were converted into the C-terminal cleavage products by breaking of the scissile bond downstream of the intein through cyclization of the catalytic block G asparagine residue, indicating that complex formation of the intein fragments had occurred but resulted in the off-pathway reaction (Fig. 2). Together, these findings underlined the improved sequence tolerance of the M86 mutant.

**Table 1:** List of Int^N^ peptides used in this study

Peptide no.	Sequence^*a*^	Splicing^*b*^ [%]		N-terminal cleavage^*c*^ [%]		Splicing^*b*^ [%]	
		WT Int^C^	M86 Int^C^	WT Int^C^ (AAA)	M86 Int^C^ (AAA)	WT Int^C^ (H73A)	M86 Int^C^ (H73A)
pep1	Fl-KKES**G**-Int^N^	61 ± 7	81 ± 1	1 ± 1	1 ± 1	0	3
pep2	Fl-KKES**A**-Int^N^	2	63 ± 1	9 ± 2	7 ± 2	0	3
pep3	Fl-KKES**Abu**-Int^N^	1	61 ± 2	18 ± 2	19 ± 3	0	4
pep4	Fl-KKES**T**-Int^N^	0	37 ± 3	31 ± 2	53 ± 4	0	21 ± 1
pep5	Fl-KKES**L**-Int^N^	0	38 ± 3	52 ± 4	80 ± 2	0	19 ± 2
pep6	Fl-KKES**H**-Int^N^	0	49 ± 2	58 ± 7	82 ± 7	0	30 ± 2
pep7	Fl-KKES**F**-Int^N^	0	44 ± 2	75 ± 2	90 ± 2	0	22 ± 3
pep8	Fl-KKES**dL**-Int^N^	0	42 ± 1	69 ± 4	100 ± 0	0	3
pep9	Fl-KKES**dF**-Int^N^	0	45 ± 0	100 ± 0	100 ± 0	0	17 ± 5

^*a*^Int^N^ = CISGDSLISLA, Fl = 5,6-carboxyfluoresceine.

^*b*^Determined by SDS-PAGE analysis.

^*c*^Determined by HPLC analysis.

### Large side chains at position (–1) can compensate for a lack of the catalytic block B histidine in the N–S acyl shift

**Fig. 3:**
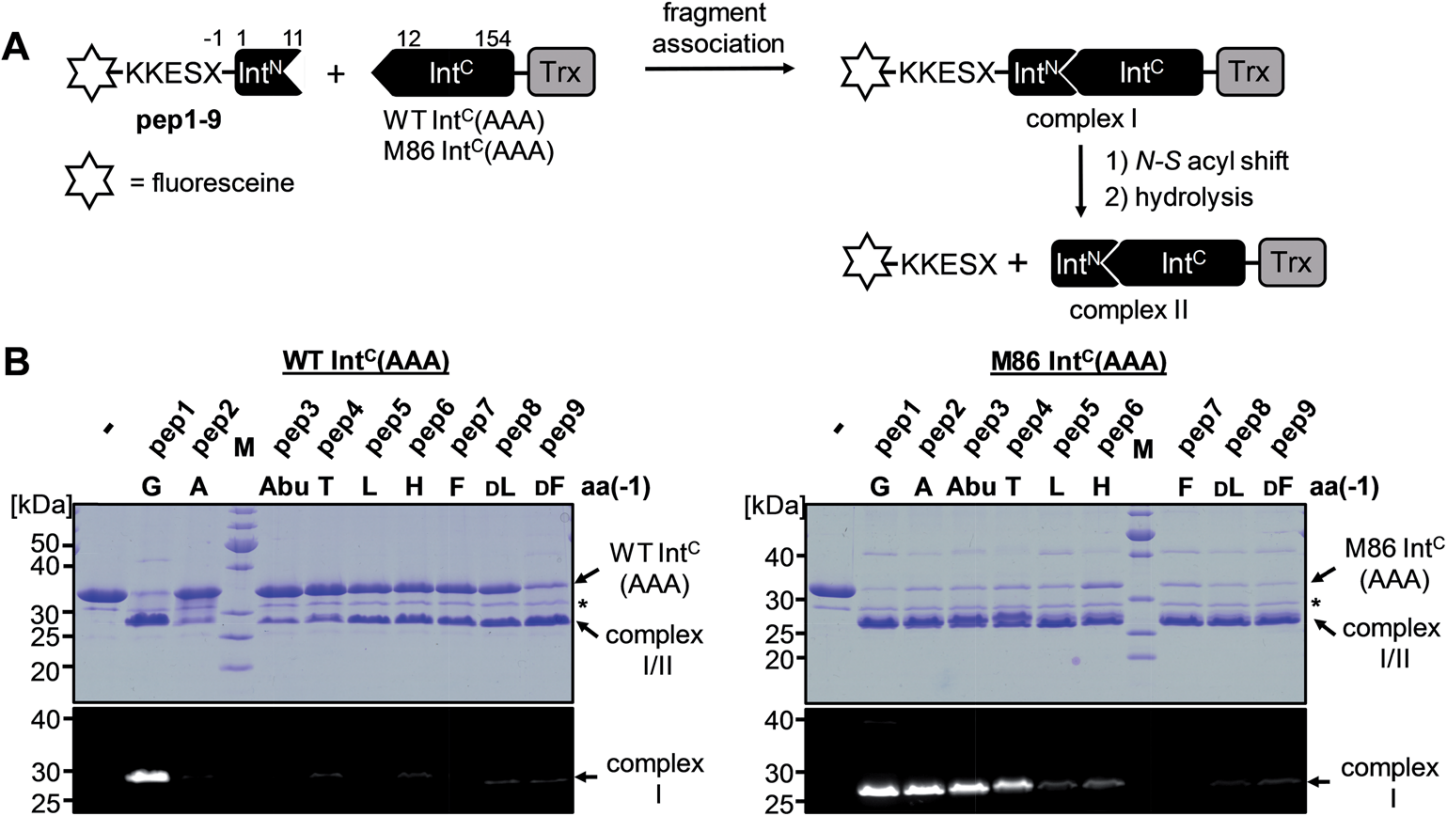
Complex formation and N-terminal cleavage of split intein pairs blocked in the essential residues of the block B and G motifs. (A) Scheme of the reactions involving the triple mutants (H73A, N154A, S+1A) of the WT *Ssp* DnaB and M86 inteins. (B) Analysis of reactions on SDS-PAGE gels using Coomassie-staining (top) or UV illumination (bottom panel). Int^C^ proteins (20 μM) were incubated with the indicated peptides (60 μM) for 24 h and then quenched by SDS loading buffer without boiling. Asterisks denote a protein contamination.

We aimed to study the association step of the intein fragments to complex formation.^24^,^30^ To this end, we incubated the Int^N^ peptides with triple mutants of the WT Int^C^ and M86 Int^C^ constructs, designed to be inactive in all three steps of the protein splicing pathway (H73A/N154A/S(+1)A, referred to as WT Int^C^(AAA) and M86 Int^C^(AAA)), to form complex I (Fig. 3A). Note that the non-covalent complex between WT Int^C^(AAA) and pep1 (Int^N^ peptide with Gly(–1)) could be observed on a Coomassie-stained denaturing SDS gel when the samples had not been boiled. The fluorescence of this complex results from the carboxyfluorescein moiety in the Ex^N^ portion of the intact pep1, as reported previously (detected under UV illumination of the gel, see second lane in lower panel in Fig. 3B).^30^ Surprisingly, a loss of fluorescence in the complex band was observed for all residues except the native Gly(–1) (pep1) in the case of the WT intein and for all larger side chains at the (–1) position (pep5 to pep9) in the case of the M86 intein (Fig. 3B, lower panels). Loss of the fluorophore by N-terminal cleavage indicated that first the peptide bond must have rearranged into the thioester, which then underwent cleavage by hydrolysis or thiolysis with DTT according to the scheme in Fig. 1B. Comparison with the band for the complex on the Coomassie-stained SDS gel indicated different yields of complex formation. In the case of the WT intein, with Ala, Abu and Thr at the flanking (–1) position (pep2 to pep4), the complexes seemed stable only to a low degree under the conditions of the SDS gel and only marginal fluorescence could be detected, possibly reflecting the lower complex yield. Peptides pep5 to pep9 harboring larger side chains at the (–1) position showed higher amounts of stable complexes; however, these were also not or only marginally fluorescent, indicating a loss of the Ex^N^ parts with the fluorophore (Fig. 3B). In the case of the M86 intein, stable complexes were observed for all peptides at nearly quantitative yields (Fig. 3B). While the assays with peptides containing Gly (pep1), Ala (pep2), or Abu (pep3) at the (–1) position yielded highly fluorescent complexes, the fluorescent signal was mostly or even nearly completely lost in the case of the larger amino acids Leu (pep5), His (pep6), Phe (pep7), d-Leu (pep8), and d-Phe (pep9) at the (–1) position. The peptide with Thr(–1) (pep4) resulted in a phenotype between these two groups. Indeed, the assay with pep4 revealed a distinct double band for the intein complex in the Coomassie-stained gel, but not under UV illumination, indicating that two different complexes were present and could be separated on the gel (Fig. 3B). A cleavage of the fluorescent Ex^N^ also results in the liberated Int^N^ part. We therefore assigned the two complexes of the Int^C^(AAA) proteins to one with an Ex^N^–Int^N^ peptide (complex I, fluorescent) and another with the remaining Int^N^ peptide (complex II, non-fluorescent). We reasoned that separation of the two complexes on the SDS-gel is possible in some cases but not necessarily in all cases.

**Fig. 4:**
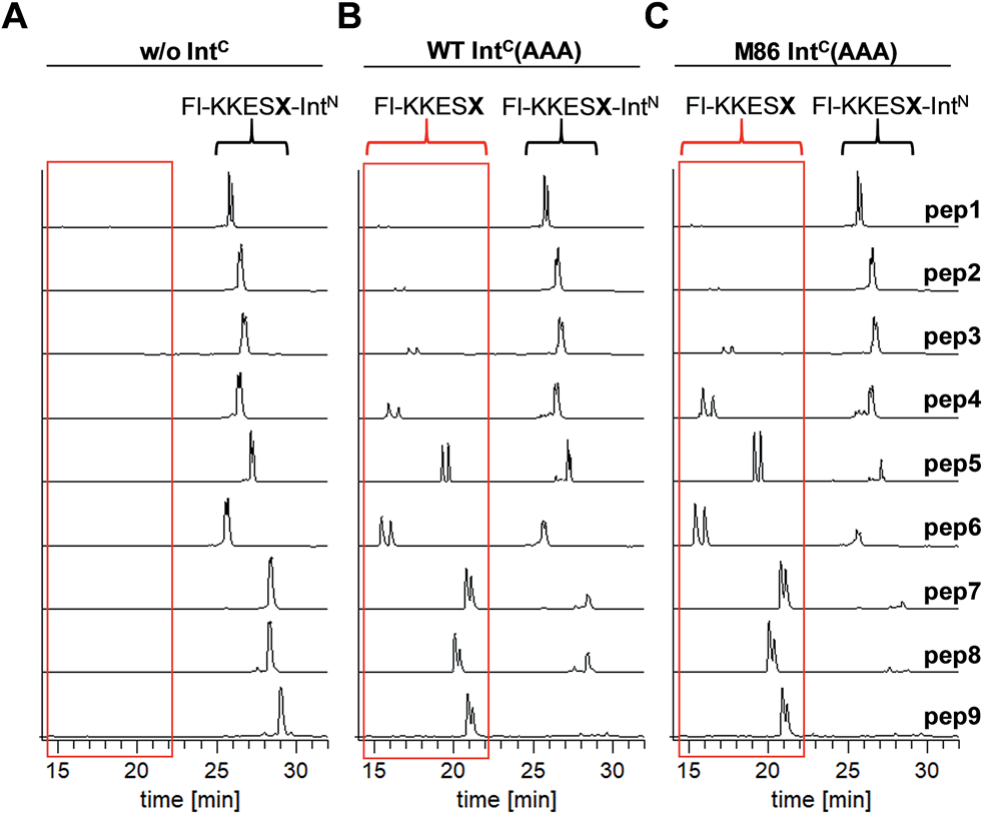
HPLC analysis of N-terminal cleavage with Int^C^ constructs containing triple mutations in the blocks B and G. Analyses of the Ex^N^–Int^N^ peptides (Fl-KKESX-Int^N^) are shown before (left panel) and after (middle and right panels) incubation with Int^C^ constructs. Absorption was measured at 280 nm, which detects only the Ex^N^ portion of the peptides (Fl-KKESX), but not the cleaved Int^N^ portion. Int^C^ proteins (15 μM) and peptides (30 μM) were incubated for 24 h. Samples were acidified with 0.1% TFA and boiled for Int^C^ precipitation prior to HPLC analysis. Note that the double peaks originate from the 5,6-isomers of the carboxyfluoresceine moiety. The identities of the cleaved Ex^N^ fragments were verified by MS-analysis (data not shown). The percentages of N-terminal cleavage are given in Table 1. The red boxes highlight the N-terminal cleavage products.

These results suggested that for the M86 intein both types of complexes were stable under the SDS conditions. In contrast, for the WT intein the complexes appeared less stable. Our observations would be consistent with all (–1) residues larger than the native Gly(–1) rendering the Ex^N^–Int^N^ complexes unstable (complex I) under these conditions and that the Coomassie-stained SDS gels only showed the cleaved complexes with the Int^N^ peptide (complex II). These differences in complex stability would be in agreement both with the higher fragment affinity of the M86 mutant in the case of the split inteins^24^ and with the higher thermostability of the *cis*-M86 intein compared to that of the *cis*-WT intein.^31^ However, since the SDS-PAGE based assays shown in Fig. 3 cannot be analyzed with sufficient quantitative accuracy, we developed an HPLC-assay to directly show and quantify the N-terminal cleavage by detecting the Ex^N^ parts (Fig. 4). While pep1 with Gly(–1) was completely stable upon incubation with the Int^C^ partners WT Int^C^(AAA) and M86 Int^C^(AAA), peptides with larger side chains at the (–1) position were cleaved at increasingly higher levels up to virtual completion (see Table 1 for quantification). These findings were consistent with our interpretation of the SDS-PAGE data. They confirmed that the block B histidine is essential for the N–S acyl shift with the native Gly(–1) residue; however, it was functionally compensated for by larger side chains at the (–1) position, in particular by those larger than Abu, both for the WT and the M86 inteins.

### The compensatory mechanism for the N–S acyl shift supports protein splicing in a block B histidine mutant

**Fig. 5:**
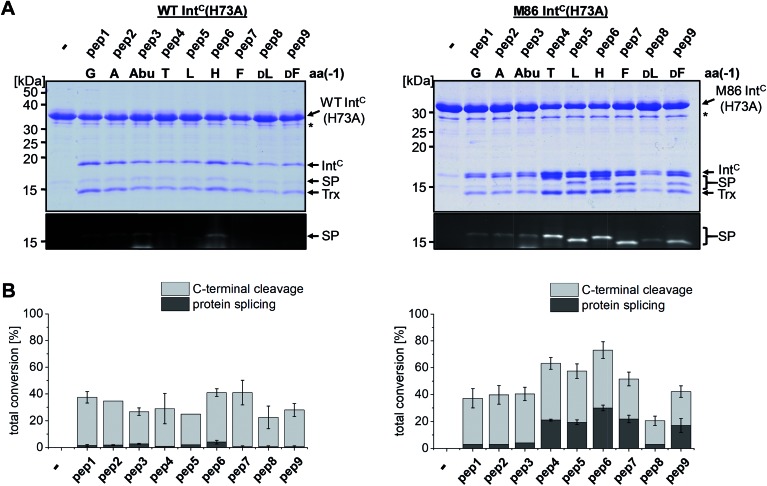
Extein dependent compensation of the block B histidine in semisynthetic protein *trans*-splicing. Reaction schemes are as shown in Fig. 1A but using the H73A mutants of both inteins. (A) Analysis of reactions on SDS-PAGE gels using Coomassie-staining (top) or UV illumination (bottom panel). Int^C^ proteins (20 μM) were incubated with the indicated peptides (60 μM) for 24 h and then quenched by SDS loading buffer and boiling. The weak bands visible for pep3 in the lower panels seem to result from a slight impurity of this peptide and were also observed in the absence of a protein partner (data not shown). (B) Yields of protein *trans*-splicing and C-terminal cleavage reactions determined by densitometric analysis of the Coomassie-stained gels shown in A, except for values for pep1–3 and pep8, which were estimated from the fluorescence signals in the bottom panels. Error-bars indicate standard deviations. Asterisks denote a protein contamination.

We next asked whether the unexpected compensatory mechanism for the otherwise essential role of the block B histidine functions only for the isolated N–S acyl shift or could also rescue the entire process of protein *trans*-splicing. This question is important as the observed effect could have been accidentally favored by a combination of the three active site substitutions in the triple mutants of the WT and M86 Int^C^ parts used to study the isolated N–S acyl shift. As expected, no splicing activity above marginal background levels was observed for the H73A single mutant of the WT intein with its native Gly(–1) residue of pep1. Also the other peptides with bulkier amino acids at the (–1) position showed only marginal yields (Fig. 5). However, large amino acids at the (–1) position did enable protein *trans*-splicing with the H73A single mutant of the M86 intein, reaching significant efficiencies of ∼20–30% yield (Fig. 5 and Table 1). Interestingly, the same side chain dependence as for the isolated N–S acyl shift was observed as splicing occurred only with (–1) residues larger than Abu. The only exception was pep8 with d-Leu at position (–1), which showed no splicing, whereas pep9 with d-Phe was active. No protein *trans*-splicing could be detected with the smaller side chains, including the native Gly(–1) in pep1 (Fig. 5). These observations further underlined our conclusion that an alternative mechanism for the N–S acyl shift, dependent on a bulky (–1) residue, must have been at play, and showed that it was compatible with the subsequent steps of protein *trans*-splicing.

### *Cis*-splicing inteins show a similar effect in response to (–1) mutations as the *trans*-splicing inteins

**Fig. 6:**
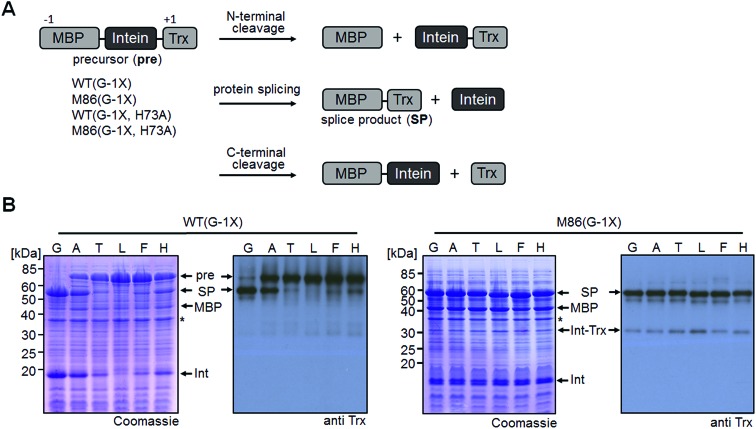
Extein dependence at the (–1) position of *cis*-inteins. (A) Reaction schemes. (B) Analysis of reactions on SDS-PAGE gels using Coomassie-staining (left panels) or Western-blotting (right panels). Shown are whole-cell lysates of *E. coli* cells expressing the *cis*-intein constructs for 3 h at 37 °C after induction with IPTG. Aliquots were mixed with SDS loading buffer and boiled.

We sought to rule out potential artefacts in the functional behavior of the mutated inteins from the association step of the artificially generated fragments. As a further control, we therefore tested the genetically accessible substitutions at the (–1) position in *cis*-variants of the WT and M86 mini-inteins. In contrast to the split WT intein, the *cis*-WT intein displayed some splicing activity with Ala(–1) and His(–1), as well as slight activity with Thr(–1) and Phe(–1) (Fig. 6), indeed suggesting a somewhat more general splice profile of the *cis*-intein that would be consistent with a more robust protein folding compared to the split intein. Nevertheless, with the exception of the Gly(–1) construct, large quantities of the intein remained as the unspliced precursors under the experimental conditions. In contrast, the M86 precursors were completely consumed and mostly converted into the splice product with a minor fraction ending up as N-terminal cleavage products (Fig. 6B).

**Fig. 7:**
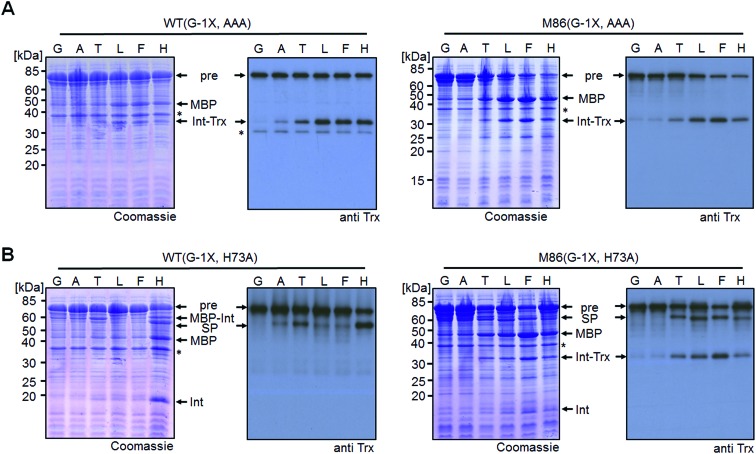
Extein dependent compensation of block B histidine of *cis*-inteins. Reaction schemes are as shown in Fig. 5A but using the H73A/N154A/S+1A mutant for the isolated N–S acyl shift resulting in N-terminal cleavage (panel A) and H73A mutants to allow for protein splicing (panel B), respectively, when the essential histidine residue is compensated for. Reactions were analyzed on SDS-PAGE gels using Coomassie-staining (left panels) or western-blotting (right panels) using whole-cell lysates of *E. coli* cells expressing the *cis*-intein constructs for 3 h at 37 °C after induction with IPTG. Aliquots were mixed with SDS loading buffer and boiled.

To address the compensatory mechanism, the *cis*-inteins with the H73A mutation were prepared with and without combination with the blocked C-terminal splice junction (H73A, N154A, S+1A = AAA mutants). For both inteins, we observed the same trend in the size dependence of the (–1) residue as found for the split inteins. In the case of the isolated N–S acyl shift, the precursors remained stable with Gly(–1), but all (–1) residues larger than alanine supported significant levels of N-terminal cleavage and hence complemented the N–S acyl shift (Fig. 7A). Furthermore, when assaying splicing in the context of the respective H73A single mutants, both inteins showed no activity with the native Gly(–1) extein residue. For the WT intein, larger residues at the (–1) position, in particular His(–1) and Thr(–1), gave rise to detectable levels of protein splicing and could therefore compensate for the missing His73 residue. This result was in contrast to that of the split WT intein and can likely be explained by a more stable folding of the *cis*-intein that prevents dissociation of the Ex^N^–Int^N^ part and helps the intein to stay on-pathway. Similarly, the M86(H73A) mutant spliced with all (–1) substitutions larger than alanine and showed yields up to ∼50% in the case of Phe(–1) (Fig. 7B). Furthermore, N-terminal cleavage was observed in these cases, suggesting that additional fractions of the proteins underwent the N–S acyl shift but then did not stay on-pathway.

### Crystal structures reveal different distortions at the N-terminal splice junction

To investigate the structural consequences of a sterically demanding side chain at the (–1) position, we crystallized the M86 intein as well as its G(–1)F and G(–1)F/H73A mutants and determined their structures at 2.0 Å, 1.5 Å, and 1.2 Å resolution, respectively (Table S2†). Similar to the available structure of the *Ssp* DnaB mini-intein at 2.0 Å,^14^ here referred to as the WT intein (PDB entry ; 1MI8), all three constructs contained the inactivating C1A and N154A mutations together with five flanking extein amino acids on both sides of the intein. The three variants of M86 crystallized in three different space groups with one or, in the case of the M86 intein itself, two copies of the protein in the asymmetric unit (Table S2†). Together with the structure of the WT intein, this allows comparing five independent intein monomers.

**Fig. 8:**
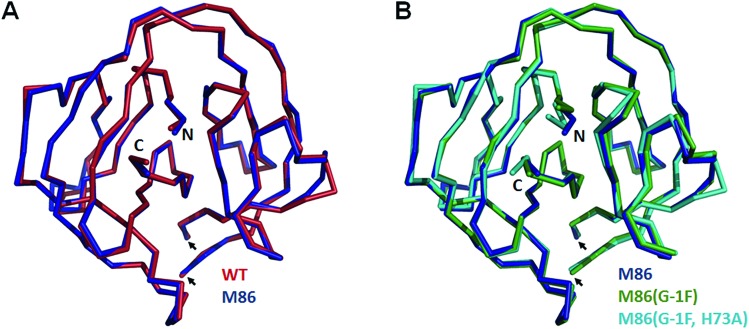
Overlay of peptide backbones of intein crystal structures. (A) Overlay of the WT *Ssp* DnaB^14^ and the M86 intein precursors. (B) Overlay of the M86 intein with its extein(–1) and active site mutants. The endonuclease loop regions between amino acids 98 and 116 (indicated with arrows) and extein residues upstream of aa(-2) and downstream of aa(+2) have been omitted for comparability. These regions are illustrated in Fig. S2.†

As expected, all of these monomers show the conserved horseshoe-like fold with nearly identical cores (RMSDs from 0.5–0.6 Å, Fig. 8 and Fig. S2†). Major differences only occurred in the flexible, solvent-exposed parts including the N- and C-terminal extein residues and the loop region covering the site of the deleted homing endonuclease domain. While this region (residue 99–115) was highly flexible in the WT intein and in three of the M86 intein monomers analyzed here, crystal contacts stabilized the respective loop in the M86(G(–1)F) mutant and allowed tracing. However, comparison to the structured parts of this loop in the other structures indicates that the flexibility of this region leads to principally different conformations, precluding further discussion (Fig. S2 and S3†).

Notably, the structures of the WT intein and of M86 as well as of its mutants are overall highly similar (RMSD = 0.6 Å), suggesting that the differences in the splicing activity must be rooted in subtle structural alterations that can only be identified after careful analysis of local geometric parameters. Since the splice site is located close to the surface of the protein and resides in different crystallographic environments, an influence of crystal contacts in the vicinity of the scissile bond would be conceivable in principle. Residues of the splice site itself are, however, not directly involved in these crystal contacts, because the five additional extein residues at both termini act as spacers that shield the splice site from neighboring molecules (Fig. S3†). It is therefore unlikely that the following observations are a consequence of different crystallographic environments.

When the WT and M86 intein are compared, slight changes of the backbone *φ*-, *ψ*- and *ω*-angles are observed at the N-terminal splice junction, which may correlate with the altered activity of the M86 mutant. The carbonyl group of the scissile peptide bond is slightly shifted between the two structures, a finding that is also reflected by differences in the *ω*-angle (Table 2). This angle is closer to 180° of the perfect *trans* conformation for the M86 intein. It is, however, unclear how significant these differences are and how they can be interpreted to explain the activity differences between the WT and M86 intein.

**Table 2:** Angles at the N-terminal scissile bond in the four DnaB intein crystal structures

Intein	Pdb-code	Aa(–1)		*ω*	Ala(1)	
		*φ*	*Ψ*		*Φ*	*Ψ*
WT *Ssp* DnaB	1MI8 (ref. 14)	–82°	–154°	174°	173°	151°
M86 (chain A)	6FRH	–102°	–176°	179°	–157°	159°
M86 (Chain B)		–101°	–177°	179°	–158°	159°
M86(G-1F)	6FRG	–125°	168°	–173°	–167°	172°
M86(G-1F, H73A)	6FRE	–124°	52°	–159°	–76°	154°

**Fig. 9:**
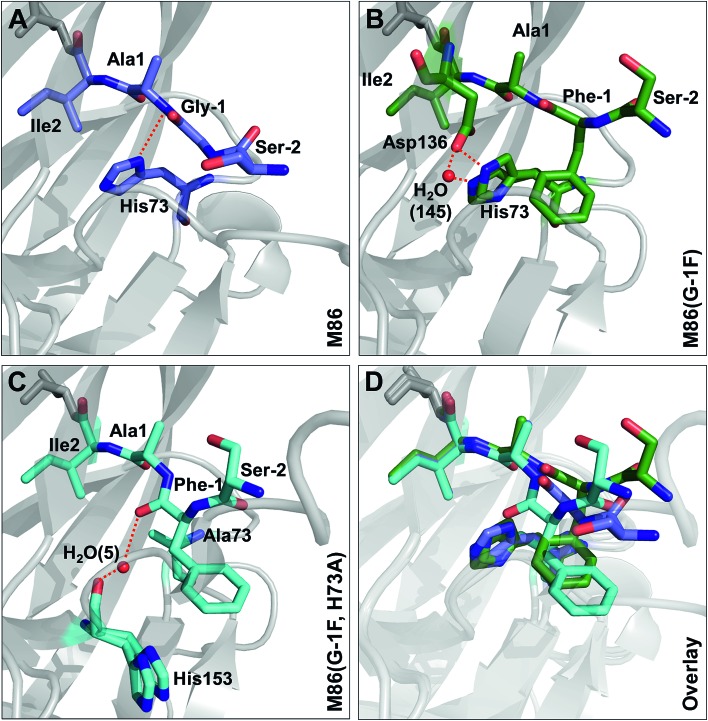
Effect of mutations on the structure of the N-terminal splice junction. Shown are close-ups of two extein residues upstream and two intein residues downstream of the scissile peptide bond. Panel C additionally shows a stick representation of the block G histidine (H153).

Comparison of the M86(G(–1)F) and M86(G(–1)F/H73A) structures with that of the M86 intein, on the other hand, revealed a few striking and obviously more significant changes at the N-terminal splice junction (Fig. 9). The sterically demanding Phe side chain in the M86(G(–1)F) mutant is partially squeezed between the His73 imidazole side chain and the N-terminal scissile bond, pushing both moieties slightly apart. The distance between the imidazole ring of the histidine side chain and the amide nitrogen of Ala1 increased from 2.9 Å in the M86 structure (with the π-nitrogen as reference point) to 3.2 or 4.3 Å in the G(–1)F mutant (δ-carbon as reference point), for which two very similar His73 conformers can be observed, indicating increased mobility of this residue (Fig. 9B). This finding suggests that the polar interaction between the δ-nitrogen of the histidine and the scissile bond amide has been disrupted, at least partially, potentially resulting in a loss of the catalytic contribution of the histidine side chain in case of the M86(G(–1)F) mutant. Consistent with this interpretation is a re-orientation of the His73 imidazole ring, which is flipped in both conformers of the G(–1)F mutant, such that its δ-nitrogen is not facing the amide nitrogen anymore (Fig. 9B). Instead, the flipped imidazole ring of one of the His73 conformers is stabilized directly by the key Asp136 residue of the block F motif, which also displays a rotated side chain relative to the M86 structure and is within the H-bonding distance of 2.9 Å to the imidazole ring. The flipped orientation of the other His73 conformer in the G(–1)F mutant is assisted by a water molecule (water 145) that is not present in the M86 structure and establishes hydrogen bonds to both the Asp136 side chain and the imidazole moiety (Fig. 9B). Importantly, the G(–1)F mutation also leads to changes within the backbone geometry around the scissile peptide bond, which expresses itself in changes of the dihedral *φ*-, *ψ*- and *ω*-angles between the (–1) position and Ala1 (Table 2). In the M86(G(–1)F) mutant, the ω-angle (–173°) shows a comparable deviation from the perfect 180° as observed for the WT intein (174°), albeit with an inverted plane. The effects are even more pronounced in the M86(G(–1)F/H73A) mutant (Table 2 and Fig. 9C). Here, the dihedral angle *φ* of the Ala1 residue is twisted to –76°, which represents a marked change of up to 91° relative to the other M86 structures. This angle is typically ∼130–170° in the other intein structures that have been crystallized with flanking Ex^N^ residues (Table S3†). The resulting twist rotates the carbonyl of the scissile peptide bond toward the space liberated by the missing His73 side chain. The new space has also accommodated a water molecule (water 5), which is not present in the other two structures. This water molecule seems to stabilize the twisted conformation by coordinating the carbonyl oxygens of Phe(–1) and of the block G His153 (Fig. 9C). Moreover, the *ψ*-angle of the Phe(–1) residue is very unusual with 52°. The overall distortion of the plane with the scissile peptide bond, described by the angle *ω* (Table 2), increases from the M86 intein (*ω* = 179°) to the G(–1)F mutant (*ω* = –173°) and the G(–1)F/H73A double mutant (*ω* = –159°). In particular the latter structure, with 21° off from the optimal *trans*-conformation, suggests that the mutations have a significant effect on the stability of the peptide bond.

## Conclusions

The dependence of inteins on their host sequence is a parameter of great importance for virtually all intein-based applications. In general, the origin of intein dependence on certain flanking amino acids is not well understood and the engineering of inteins with greater or unlimited tolerance is challenging.^24^,^32^–^34^ The M86 mutant of the *Ssp* DnaB intein (here referred to as WT intein) was selected by sequential directed evolution for its more general activity and is one of the few known intein mutants with improved extein promiscuity.^24^,^27^ This study reports the first systematic investigation on various residues at the upstream flanking (–1) position, which naturally is a Gly residue, of both the M86 mutant and the parent intein. Our data confirmed the general activity of the M86 intein and its clear superiority over the parent intein. Importantly, whereas the WT intein showed no or only very little activity with residues larger than Ala, the M86 mutant was able to splice efficiently with all tested amino acids, both *in cis* and *in trans*.

While it has already been well-established that the nature of the flanking extein amino acids can influence the efficiency of the protein splicing reaction, there is little precedent for non-native extein residues to promote, instead of to impair, the protein splicing pathway.^35^,^36^ Furthermore, to our knowledge, no such case has been reported in which the loss of a catalytically critical residue has been compensated for by extein optimization, which would indicate a direct functional contribution. As a surprising finding during this study we discovered that substitutions at the (–1) residue enabled both the WT and the M86 intein to catalyze the N–S acyl shift in the context of a H73A/N154A/S+1A mutant, which was designed to block all steps of the splicing pathway. The histidine of the block B motif is essential for this first step in the splicing pathway in the native sequence context (Fig. 1C). The functional complementation of the histidine correlated with the size of the (–1) side chain (Fig. 1D) and became significant with side chains larger than Ala or Abu. While it is conceivable for several of our selected amino acids to help catalyze the N–S acyl shift through their side chain, *e.g.* Thr, His and Phe, the aliphatic Leu side chain also promoted this effect, thus ruling out the requirement of electronic effects. The importance of steric demand was confirmed by the crystal structure of the M86 precursor intein with the G(–1)F mutation and revealed a constraint imposed around the scissile bond. The increased distance of the His73 side chain from the scissile bond indicated that the steric demand at the (–1) position may uncouple His73 from its role in the natural mechanism of the N–S acyl shift, at least in part. Strikingly, the functional complementation of the catalytic His73 through a large (–1) side chain also extended to a single H73A mutant of the M86 intein. Here, splicing activity was restored, whereas the same intein with the native Gly(–1) was completely inactive. This finding showed that the subsequent steps in the protein splicing pathway are not critically impaired, neither by introducing a larger amino acid at position (–1) nor by substituting the catalytic His73, suggesting only local perturbations that do not affect the remaining configuration of the active site. Again, this hypothesis was confirmed by the crystal structure of the G(–1)F/H73A mutant, which revealed that significant changes are indeed restricted to the N-terminal splice junction. The functional complementation of His73 by large (–1) side chains can be seen as evidence to rule out the critical role of this block B residue in deprotonating Cys1 or in other steps of the pathway; however, an increased ground-state destabilization by the G(–1)F mutations might also obviate the need for His73 as a general base. These conclusions are in agreement with an earlier study in which we showed that a single *N*-methyl group at the catalytic Cys1 could also functionally compensate for a mutated His73 side chain.^10^ Thus, in addition to the different natural mechanisms to promote the N–S acyl shift that have been recognized so far (see Introduction), these two engineered compensatory mechanisms for the N–S acyl shift further underline the functional plasticity of the active site and support the idea that many different catalytic solutions can serve in protein splicing.

Our crystal structures also support the model that the first reaction in protein splicing is largely brought about by ground-state destabilization through distortion around the scissile peptide bond. The structures of the M86(G(–1)F) and M86(G(–1)F/H73A) mutants revealed snapshots in which the (–1) and 1 residues adopt unusual geometries (Table 2). The significant differences at the N-terminal splice junction in these two structures indicate that the N–S acyl shift can be brought about by different, non-native distortion strategies. A recent study used DFT calculations to underline the fact that the rate of the N–S acyl shift is favored in the *cis* over the *trans* conformation of the scissile bond in a model amide because of the easier approach of the nucleophile to the carbonyl group.^37^ However, the situation in the intein's active site is more complicated because residues around the scissile bond will shape the conformation and polarization of the peptide chain, participating bonds and groups through steric and electronic contacts. Therefore, the optimal conformation for reaching the transition state may not be possible. We assume that at least one more type of conformational distortion, in addition to those approached by our crystal structures, can support non-native compensatory mechanisms, since our data showed that d-amino acids at the (–1) position compensated for the block B histidine as well (Fig. 1D). A diastereoisomer at the N-terminal splice junction would be expected to have a different influence along these lines.^38^ Thus, the scaffold of the inteins' active site appears to provide a confined space in which strains beneficial to the N–S acyl shift can be exerted on the scissile bond. This space displays functional plasticity for different catalytic solutions. An important residue to hold the peptide in this space seems to be the conserved and well-characterized Thr from the block B motif, which was described to spring-load the N-terminal splice junction in the *Ssp* DnaE intein (corresponding to Thr70 in the WT and M86 variants of the *Ssp* DnaB intein).^39^ Indeed, the position of this active site residue is virtually identical for all four structures compared here (not shown). Furthermore, additional catalytic residues such as the (+1) nucleophilic side chain serve to remove the (thio)ester from the equilibrium through the subsequent steps of the protein splicing pathway.

Notably, large side chains at the (–1) positions do occur in many native inteins.^40^ It is therefore tempting to speculate that the mechanism discovered here also contributes or is even crucial to catalysis in some of those cases. Examples from the literature that may hint at this possibility are the *Pho* RadA intein and the *Tko* CDC21-1 intein orthologs. The former intein has a lysine at position (–1) and lower levels of splice product were observed when this residue was replaced with smaller amino acids such as alanine or glycine.^34^ The latter constitute a small group of inteins naturally lacking the block B histidine residue and were found to have evolved a compensatory mechanism to better stabilize the oxyanion;^16^ however, they also come with a bulky leucine at position (–1). Such inteins could have originated from a parent intein in which the histidine was accidentally lost by spontaneous mutation. They could have remained sufficiently active due to the compensatory mechanism we describe here, followed by further optimization of the alternative catalytic strategy during evolution. Our results suggest this to be a viable possibility for protein engineering attempts, in particular to adapt inteins to very large, possibly unnatural flanking moieties, although it is at present unpredictable which inteins would be favorably amenable to such an approach and what its limitations would be.

In summary, we have further investigated the high sequence promiscuity of the M86 intein and reported its crystal structure. We have discovered and structurally corroborated a new extein-dependent mechanism to promote the N–S acyl shift through distortion around the scissile peptide bond independent of the block B histidine that might also be used by native inteins. These results underline the catalytic flexibility of inteins and may be useful for future attempts to engineer inteins with high sequence tolerance.

## Experimental section

### Materials and methods

Synthetic oligonucleotides were ordered from Biolegio. Restriction enzymes were obtained from Fermentas. Buffer reagents, antibiotics and media components were purchased from Carl Roth, Applichem, Sigma Aldrich or Thermo Scientific. Ni-NTA agarose was ordered from Cube Biotech. Rabbit-derived anti-Trx antibody was purchased from Sigma-Aldrich and used in 1 : 10 000 dilution. Porcine derived anti-rabbit antibody was purchased from Dako and used in 1 : 5000 dilution. Error bars represent standard deviations from at least three independent experiments.

### Protein expression and purification

*E. coli* BL21 Gold (DE3) cells were transformed with the respective plasmids (Table S4†). Cells were cultured at 37 °C in LB medium containing the corresponding antibiotics (ampicillin: 100 μg ml^–1^ or kanamycin: 50 μg ml^–1^). Upon reaching an OD_600_ of 0.6–0.8, protein expression was induced by IPTG addition (0.4 mM) and carried out for 4 h at 28 °C. Cells were harvested by centrifugation and the cell pellets were resuspended in the respective purification buffer. All His_6_-tagged proteins were purified under denaturing conditions (50 mM Tris/HCl, 300 mM NaCl, 8 M urea, pH8). Denaturing purification conditions were not strictly required but yielded higher protein amounts and improved protein purity. Cell lysis under denaturing conditions was performed using a Potter-Elvehjem homogenizer followed by protein purification *via* nickel affinity chromatography. Protein renaturation was carried out by dialysis against splice buffer (50 mM Tris/HCl, 300 mM NaCl, 1 mM EDTA, pH7). For protein crystallization, cells were lysed under native conditions using an emulsifier (Avestin EmulsiFlex®-C5 high-pressure emulsifier). Purification was performed *via* a C-terminal *Ssp* GyrB(1-150)-CBD-Tag, which allowed purification *via* chitin affinity chromatography followed by DTT induced Tag removal.^10^,^41^ Protein concentration was determined *via* absorption spectroscopy at 280 nm using the calculated extinction coefficients. For crystallization purposes, the M86 (G-1F) and (G-1F, H73A) mutant inteins were subjected to an additional size exclusion chromatography to separate aggregates. The proteins were loaded onto a HiLoad™ 75 26/600 Superdex™ (GE Healthcare) column connected to an ÄKTA purifier FPLC-system (GE Healthcare) and eluted with 50 mM Tris/HCl, 100 mM NaCl pH 7.6. Fractions containing pure protein were collected, concentrated to 31 mg ml^–1^, flash frozen in liquid nitrogen and stored at –80 °C.

### Peptide synthesis and purification

Peptides were synthesized by standard Fmoc solid phase peptide synthesis (SPPS) using a Liberty microwave-assisted peptide synthesizer (CEM, Kamp-Lintford, Germany) and purified by HPLC according to previously published protocols,^10^,^27^ except for replacing hydroxybenzotriazole (HOBt) with ethyl cyano(hydroxyimino)acetate (Oxyma Pure).

### Protein *trans*-splicing assay

Protein *trans*-splicing was performed in splice buffer at 25 °C. The reaction was initiated by mixing the N-terminal and the C-terminal intein fragments at the indicated concentration in the presence of 2 mM TCEP. The reaction was stopped by addition of 4× SDS-PAGE loading buffer (500 mM Tris/HCl, 8% (w/v) SDS, 40% (v/v) glycerine, 20% (v/v) β-mercaptoethanol, 5 mg L^–1^ bromophenol blue, pH 6.8) and subsequent boiling (10 min, 95 °C).

### N–S acyl shift assay *in trans*

*Trans*-N–S-acyl-shift assays were performed in splice buffer at 25 °C. The reaction was initiated by mixing the N-terminal and the C-terminal intein fragments at the indicated concentration in the presence of 2 mM TCEP or, alternatively, in the presence of 2 mM DTT in the case of HPLC analysis. For SDS-PAGE analysis, the reaction was stopped by addition of 4× SDS-PAGE loading buffer without boiling to keep the intein complex intact. For HPLC analysis, the reaction was stopped by addition of 0.1% TFA and subsequent boiling (10 min, 95 °C) to precipitate protein components. A linear gradient from 10% to 50% ACN within 35 min was used (buffer A: ddH_2_O + 0.1% TFA; buffer B: ACN + 0.1% TFA; C18 column: Agilent, ZORBAX SB-C18 (50 × 3 mm, 80 Å, 1.8 μm)). Peak identities were determined by ESI/MS.

### Protein splicing and N–S acyl shift assay *in cis*

*E. coli* BL21 Gold (DE3) cells harboring the respective expression plasmids were cultured at a 2 ml scale at 37 °C. Upon reaching OD_600_ 0.6–0.8, expression was induced by IPTG (0.4 mM) addition. After 3 h at 37 °C, the cells were harvested by centrifugation. The cell pellet was resuspended in ddH_2_O, mixed with 4× SDS-PAGE loading buffer and boiled (15 min, 95 °C) prior to SDS-PAGE analysis.

### Protein crystallization

All crystallization experiments were performed at 20 °C. For the M86 intein, a grid screen based on previously published crystallization conditions for WT *Ssp* DnaB (PDB-ID: ; 1MI8)^14^ was performed, using the hanging drop vapor diffusion method. Well-diffracting crystals grew in a 2 μl drop consisting of an equal volume of protein (15 mg ml^–1^ in 20 mM Tris/HCl, 250 mM NaCl, 1 mM EDTA, pH 8.5) and reservoir solution (0.1 M Tris pH 7.6, 22% (w/v) PEG4000) equilibrated against 500 μl reservoir solution. The crystals were cryoprotected in a reservoir solution supplemented with 20% (v/v) glycerol. Crystallization experiments for the M86 (G-1F) and M86 (G-1F, H73A) inteins were carried out with the sitting drop vapor diffusion method. The crystallization drops consisted of 0.2 μl of the respective M86 mutant (25 mg ml^–1^ in 50 mM Tris/HCl pH 7.6, 100 mM NaCl) and an equal volume of reservoir solution and were equilibrated against 60 μl reservoir solution. All plates were set up with a Honeybee 961 dispensing robot (Zinsser Analytic). After initial crystallization conditions were identified using the JCSG Core suites I-IV (Qiagen), they were optimized in grid screen format. The reservoir solutions for this were prepared with a Formulator liquid handling system (Formulatrix). Well-diffracting crystals of M86(G-1F) grew after combining the protein with 0.1 M Na_3_ citrate pH 5.3, 48% (w/v) PEG200. The crystals were already cryoprotected from the precipitant. After grid screen optimization of the M86 (G-1F, H73A) mutant, a Silver Bullets™ (Hampton Research) additive screen was performed to improve the crystal quality. The additive screen was mixed with the optimized precipitant (0.1 M HEPES pH 7.5, 10% (w/v) PEG8000) in a ratio of 1 : 10. Finally, well-diffracting crystals were obtained in 0.1 M HEPES pH 7.5, 10% (w/v) PEG8000 supplemented with 0.1% (w/v) protamine sulfate and 2 mM HEPES pH 6.8. The M86(G-1F, H73A) crystals were cryoprotected supplementing the reservoir solution with 10% (v/v) (2*R*,3*R*)–2,3-butanediol. All crystals were flash-cooled in liquid nitrogen before data collection.

### Data collection, processing, phasing and refinement

Diffraction data were measured at the Swiss Light Source (Paul Scherrer Institute, Villigen, Switzerland) at beamlines X06DA and X10SA at 100 K. The data were integrated and indexed with XDS^42^ and scaled with Aimless^43^ from the CCP4 suite.^44^ The structures were phased by molecular replacement with Phaser^45^ from the PHENIX suite^46^ using WT *Ssp* DnaB (PDB-ID: ; 1MI8)^14^ as the search model. The initial models were refined in iterative cycles of manual refinement with COOT^47^ and automated refinement with phenix.refine.^46^,^48^ The structures were validated with MolProbity^49^ and deposited in the Protein Data Bank^50^ with accession codes ; 6FRH (M86), ; 6FRG (M86(G-1F)) and ; 6FRE (M86(G-1, H73A)). Full data collection and refinement statistics are shown in Table S2.†

## Conflicts of interest

There are no conflicts to declare.

## Supplementary Material

Supplementary information
