# Supplementary material for: A functional interplay between intein and extein sequences in protein splicing compensates for the essential block B histidine
Source: Chem Sci. 2018 Oct 3;10(1):239–51. doi: 10.1039/c8sc01074a (PMC6333167; doi:10.1039/c8sc01074a)
Supplement: Supplementary file 1 [file SC-010-C8SC01074A-s001.pdf]

Electronic Supplementary Material (ESI) for Chemical Science.
This journal is © The Royal Society of Chemistry 2018

SUPPORTING INFORMATION

A functional interplay between intein and extein sequence in protein splicing compensates for the essential block B histidine

Kristina Friedel[1], Monika Popp[2], Julian C. J. Matern[1], Emerich M. Gazdag[2], Ilka V. Thiel[1], Gerrit Volkmann[1], Wulf Blankenfeldt[2,3], Henning D. Mootz*

[1]Institute of Biochemistry
University of Muenster
Wilhelm-Klemm-Str. 2
48149 Münster, Germany

[2]Structure and Function of Proteins
Helmholtz Centre for Infection Research
Inhoffenstraße 7
38124 Braunschweig, Germany

[3]Institute for Biochemistry, Biotechnology and Bioinformatics,
Technische Universität Braunschweig
Spielmannstraße 7
38106 Braunschweig, Germany

* to whom correspondence should be addressed:
E-mail: Henning.Mootz@uni-muenster.de

Supporting Tables

Table S1. Rates of *trans*-splicing

	k_{SP} [$10^{-4}s^{-1}$]	
	**M86 Int^{C}**	**M86 Int^{C}(H73A)**
**pep1**	13.3 ± 0.9	-
**pep2**	4.1 ± 0.3	-
**pep3**	4.3 ± 0.3	-
**pep4**	5.7 ± 0.7	0.31 ± 0.06
**pep5**	6.5 ± 0.6	0.33 ± 0.04
**pep6**	14.8 ± 1.9	0.74 ± 0.11
**pep7**	22.8 ± 2.3	0.40 ± 0.04
**pep8**	2.0 ± 0.3	-
**pep9**	20.0 ± 5.3	0.25 ± 0.05

Table S2: Data collection, processing and refinement statistics of M86 mutants. Values in parentheses are for the highest resolution shell.

Dataset	M86	M86 (G-1F)	M86 (G-1F, H73A)
**data collection and processing**			
Beamline[¥]	X10SA, SLS	X06DA, SLS	X06DA, SLS
Detector	Pilatus 6M	Pilatus 2MF	Pilatus 2MF
Wavelength (Å)	0.9793	1	1
Resolution range (Å)	37.05-2.03 (2.08-2.03)	43.94-1.54 (1.56-1.54)	48.05-1.22 (1.24-1.22)
Space group	P1	$P4_222$	$P4_322$
Cell dimensions			
a, b, c (Å)	34.6, 40.9, 57.9	71.5, 71.5, 87,8	52.6, 52.6, 118.4
α, β, γ (°)	84.4, 83.5, 65.0	90, 90, 90	90, 90, 90
Mosaicity(°)[#]	0.28	0.11	0.07
Total reflections	62054 (4704)	547813 (27493)	2553000 (119585)
Unique reflections	18028 (1329)	34745 (1683)	49184 (2332)
Mean I/σ (I)	5.8 (1.9)	24.5 (2.0)	28.6 (2.0)
Multiplicity	3.4 (3.5)	15.8 (16.3)	51.9 (51.3)
Completeness (%)	96.8 (96.2)	100.0 (100.0)	98.1 (95.8)
R_{meas} (%)[§]	18.3 (89.7)	6.8 (170.1)	9.5 (321.2)
R_{pim} (%)[$]	9.8 (47.5)	2.3 (58.1)	1.8 (60.4)
$CC_{1/2}$[+]	98.2 (70.6)	100.0 (64.6)	100.0 (69.9)
Wilson B-factor (Å^2)	20.5	18.4	12.7
**refinement**			
Resolution range (Å)	31.95-2.03	43.94-1.53	48.05-1.22
R_{work} (%)	22.2	14.3	17.5
R_{free} (%)	24.2	17.0	18.8
No. of non-hydrogen atoms			
Protein	2394	1291	1317
Ligand	-	70	-
Water	177	221	248
R.m.s. deviations			
Bonds (Å)	0.002	0.018	0.006
Angles (°)	0.492	1.669	0.934
Average B-factors (Å^2)			
Protein	31.93	26	22
Ligand	-	63	-
Water	34.96	44	35
Ramachandran plot (%)			
Favored regions	98.0	97.6	98.0
Outliers	0	0	0
MolProbity score[‡]	0.98	1.61	1.12
molecules/ASU	2	1	1
PDB code	6FRH	6FRG	6FRE

[¥] SLS: Swiss Light Source (Paul Scherrer Institute, Villigen, Switzerland).
[#] Value as reported by *AIMLESS.*[1]
[+] $CC_{1/2}$ Correlation coefficient between the intensities of two random half data sets.[2]
[§] $R_{meas} = \Sigma_{hkl} (N/(N-1))^{1/2} \Sigma_i | I_i(hkl) - \langle I(hkl)\rangle | / \Sigma_{hkl} \Sigma_i I_i(hkl)$, where N is the number of observations of the reflection with index hkl and I_i is the intensity of its ith observation.
[$] $R_{pim} = \Sigma_{hkl} (1/(N-1))^{1/2} \Sigma_i | I_i(hkl) - \langle I(hkl)\rangle | / \Sigma_{hkl} \Sigma_i I_i(hkl)$ where N is the multiplicity.[3]
[‡] Value as reported by *MolProbity.*[4]

Table S3. Selected angles and sequences of all available intein crystal structures including flanking residue*

Intein	Pdb[Ref]	native N-Extein (5aa)	N-Extein	Mutation within intein	C-Extein	native C-Extein (5aa)	τ ($C_{\alpha,-1}$-C'-N)	φ(aa(-1))	ψ(aa(-1))	ω_1	φ(aa(1))	ψ(aa(1))	τ ($C_{\alpha,1}$-C'-N)
Mja KlbA	2JMZ[5]	TGHDG-	MNTGHDG-	N(G:7)A	-**S**SGTLHHHHHH	-CSGTL	112.9°	-93.0°	-169.0°	165.2°	-177.4°	170.8°	107.7°
Mxe GyrA	1AM2[6]	AAMRY-	A-	C(A:1)A	-	-TEAPL	104.9°	-	137.1°	0.7°	-136.8°	164.8°	109.2°
Npu DnaB	4O1R[7]	LRESG-	SGG-	C(A:1)A	-	-SIEQD	113.7°<br>112.6°	-88.5°<br>113.3°	171.2°<br>176.0°	172.1°<br>-170.7°	-133.7°<br>-138.1°	176.6°	110.7°
Npu DnaE	2KEQ[8]	KFAEY-	GG-	C(A:1)A	-	-CFNKS	113.8°	60.2°	-167.0°	172.6°	-158.3°	171.6°	112.2°
Npu DnaE	4KL5[9]	KFAEY-	SGG-	C(A:1)A	-**A**DNG	-CFNKS	113.3°	66.7°	-162.3	171.9°	-169.6°	171.1°	111.4°
Npu DnaE$_{split}$	4LX3[10]	KFAEY-	HHHHHH-	-	-	-CFNKS	102.9°	-137.6°	151.0°	-144.2°	-164.5°	169.7°	111.1°
Pho RadA	4E2U[11]	FGSGK-	SQHM-	C(A:1)A	-**A**Q	-TQLAH	110.3°	-117.5°	145.7°	-174.4°	-140.4°	-35.4°	116.5°
Sce VMAI	1EF0[12]	IIYVG-	MKAEEGKLEG-	C(A:1)A, N(G:7)A	-CGER	-CGERG	108.3°<br>99.1°	-94.2°<br>-121.2°	139.5°<br>109.2°	-179.5°<br>-177.2	-157.6°<br>-109.0°	173.6°<br>124.7°	113.5°<br>115.0°
Sce VMAI	1JVA[13]	IIYVG-	MSNSDAIIYVG-	C(A:1)S, H(B:10)N, N(G:7)S	-**S**GERGNEMAE	-CGERG	112.3°<br>110.8°	-61.9°<br>-62.1°	150.7°<br>144.1°	179.7°<br>-179.1°	-166.3°<br>-148.8°	-177.0°<br>-173.7°	112.9°<br>111.8°
Sce VMAI	1GPP[14]	IIYVG-	MHHHHHHGSA-	-	-	-CGERG	109.8°	-95.2°	122.9°	-170.3°	-130.4	125.4	113.9°
Ssp DnaB	1MI8[15]	LRESG-	SG-	C(A:1)A, N(G:7)A	-SI	-SIEQD	110.7°	-82.0°	-153.6°	174.4°	173.0°	150.9°	109.5°
M86	6FRH	LRESG-	MLRESG-	C(A:1)A, N(G:7)A	-SIEQDKLGG	-SIEQD	112.6°<br>112.6°	-102.0°<br>-101.3	-176.1°<br>-176.7°	178.9°<br>178.5°	-157.0°<br>-157.9°	158.7°<br>158.8°	108.5°<br>108.5°
M86(G-1F)	6FRG	LRESG-	MLRESG-	C(A:1)A, N(G:7)A, G(-1)F	-SIEQDKLGG	-SIEQD	106.7°	-125.0°	168.1°	-173.3°	-167.0°	171.6°	111.6°
M86(G-1F, H73A)	6FRE	LRESG-	MLRESG-	C(A:1)A, N(G:7)A, G(-1)F,<br>H(B:10)A	-SIEQDKLGG	-SIEQD	107.0°	-124.0°	52.2°	-158.6°	-76.5°	153.9°	114.1°
Ssp DnaE	1ZDE[16]	KFAEY-	IIAMEKFAEY-	C(A:1)A, N(G:7)A,	-CFNISTGP	-CFNKS	111.2°	-64.3°	165.8°	-179.2°	-158.0°	159.1°	112.2°
Ssp DnaE$_{redox-trapped}$	3NZM[17]	KFAEY-	KSPDPFCPG-	-	-	-CFNKS	114.5°	-123.5°	68.0°	179,1	-127.3°	159.9°	110.4°
Ssp DnaE(T69A)	4GIG[18]	KFAEY-	KSPDPFCPG-	T(B:7)A	-	-CFNKS	115.6°	-91.9°	33.7°	179.2°	-101.1°	154.4°	112.0°
Tvo VMA	4O1S[7]	FGSGK-	SGGK-	C(A:1)A	-**A**	-TVIQH	110.8°<br>108.9°	-75.6°<br>-69.0°	126.6°<br>116.5°	179.6°<br>-179.6°	-159.4°<br>-155.1°	170.3°<br>171.5°	112.0°<br>110.0°

* a recent paper reporting *Ssp* DnaE structures was not included in the analysis because the extein residues were arranged for cyclization and are therefore likely to be in a highly strained conformation.[19]

Table S4. Expression plasmids and amino acid sequences

Protein	Reference	Expression plasmid	Vector backbone	Sequence (intein sequence underlined)
WT Int^C-Trx-His_6	Ref[20]	pCL20	pSU38	MGTSSTGKRVSIKDLLDEKDFEIWAINEQTMKLESAK<br>VSRVFCTGKKLVYILKTRLGRTIKATANHRFLTIDGWK<br>RLDELSLKEHIALPRKLESSSLQLSPEIEKLSQSDIYWDS<br>IVSITETGVEEVFDLTVPGPHNFVANDIIVHNSIEGSGG<br>GSDKIIHLTDDSFDTDVLKADGAILVDFWAHWCGPCK<br>MIAPILDEIADEYQGKLTVAKLNIDHNPGTAPKYGIRGI<br>PTLLLFKNGEVAATKVGALSKGQLKEFLDANLAGSGS<br>RSHHHHHH
WT Int^C(H73A)-Trx-His_6	Ref[21]	pJB04	pSU38	WT Int^C with H73A mutation
WT Int^C(H73A, N154A, S+1A))-Trx-His_6	This work	pPJ03	pSU38	WT Int^C with H73A, N154A, S+1A mutation
M86 Int^C-Trx-His_6	Ref[22]	pIT21	pET16b	MGTSSTGKRVPIKDLLGEKDFEIWAINEQTMKLESAK<br>VSRVFCTGKKLVYTLKTRLGRTIKATANHRFLTIDGW<br>KRLDELSLKEHIALPRKLESSSLQLAPEIEKLPQSDIYW<br>DPIVSITETGVEEVFDLTVPGLRNFVANDIIVHNSIEGSG<br>GGSDKIIHLTDDSFDTDVLKADGAILVDFWAHWCGPC<br>KMIAPILDEIADEYQGKLTVAKLNIDHNPGTAPKYGIR<br>GIPTLLLFKNGEVAATKVGALSKGQLKEFLDANLAGS<br>VDRSHHHHHH
M86Int^C(H73A)-Trx-His_6	This work	pKF14	pET16b	M86 Int^C with H73A mutation
M86 Int^C(H73A, N154A, S+1A))-Trx-His_6	Ref[22]	pIT28	pET16b	M86 Int^C with H73A, N154A, S+1A mutation
MBP-WT-Trx	This work	pKF18	pMST[23]	MKTEEGKLVIWINGDKGYNGLAEVGKKFEKDTGIKVT<br>VEHPDKLEEKFPQVAATGDGPDIIFWAHDRFGGYAQS<br>GLLAEITPDKAFQDKLYPFTWDAVRYNGKLIAYPIAVE<br>ALSLIYNKDLLPNPPKTWEEIPALDKELKAKGKSALMF<br>NLQEPYFTWPLIAADGGYAFKYENGKYDIKDVGVDN<br>AGAKAGLTFLVDLIKNKHMNADTDYSIAEAAFNKGET<br>AMTINGPWAWSNIDTSKVNYGVTVLPTFKGQPSKPFV<br>GVLSAGINAASPNKELAKEFLENYLLTDEGLEAVNKD<br>KPLGAVALKSYEEELAKDPRIAATMENAQKGEIMPNIP<br>QMSAFWYAVRTAVINAASGRQTVDEALKDAQTNSSS<br>NNNNNNNNNNLGIEGRGTLESGCISGDSLISLASTGKR<br>VSIKDLLDEKDFEIWAINEQTMKLESAKVSRVFCTGKK<br>LVYILKTRLGRTIKATANHRFLTIDGWKRLDELSLKEH<br>IALPRKLESSSLQLSPEIEKLSQSDIYWDSIVSITETGVEE<br>VFDLTVPGPHNFVANDIIVHNSIEGSGGTGMSDKIIHLT<br>DDSFDTDVLKADGAILVDFWAEWCGPCKMIAPILDEI<br>ADEYQGKLTVAKLNIDQNPGTAPKYGIRGIPTLLLFKN<br>GEVAATKVGALSKGQLKEFLDANLA
MBP-WT(H73A)-Trx	This work	pKF28	pMST	WT intein with H73A mutation
MBP-WT(H73A, N154A, S+1A)-Trx	This work	pKF23	pMST	WT intein with H73A, N154A, S+1A mutation
MBP-M86-Trx	Ref[24]	pAba12	pMST	MKTEEGKLVIWINGDKGYNGLAEVGKKFEKDTGIKVT<br>VEHPDKLEEKFPQVAATGDGPDIIFWAHDRFGGYAQS<br>GLLAEITPDKAFQDKLYPFTWDAVRYNGKLIAYPIAVE<br>ALSLIYNKDLLPNPPKTWEEIPALDKELKAKGKSALMF<br>NLQEPYFTWPLIAADGGYAFKYENGKYDIKDVGVDN<br>AGAKAGLTFLVDLIKNKHMNADTDYSIAEAAFNKGET<br>AMTINGPWAWSNIDTSKVNYGVTVLPTFKGQPSKPFV<br>GVLSAGINAASPNKELAKEFLENYLLTDEGLEAVNKD<br>KPLGAVALKSYEEELAKDPRIAATMENAQKGEIMPNIP<br>QMSAFWYAVRTAVINAASGRQTVDEALKDAQTNSSS<br>NNNNNNNNNNLGIEGRGTLESGCISGDSLISLASTGKR<br>VPIKDLLGEKDFEIWAINEQTMKLESAKVSRVFCTGKK<br>LVYTLKTRLGRTIKATANHRFLTIDGWKRLDELSLKEH<br>IALPRKLESSSLQLAPEIEKLPQSDIYWDPIVSITETGVE<br>EVFDLTVPGLRNFVANDIIVHNSIEGSGGTGMSDKIIHL<br>TDDSFDTDVLKADGAILVDFWAEWCGPCKMIAPILD<br>EIADEYQGKLTVAKLNIDQNPGTAPKYGIRGIPTLLLFK<br>NGEVAATKVGALSKGQLKEFLDANLA
MBP-M86(H73A)-Trx	This work	pKF21	pMST	M86 intein with H73A mutation
MBP-M86(H73A, N154A, S+1A)-Trx	This work	pKF27	pMST	M86 intein with H73A, N154A, S+1A mutation
MBP-WT(G-1A)-Trx	This work	pKF190	pMST	WT intein with G-1A mutation
MBP-WT(G-1A, H73A)-Trx	This work	pKF193	pMST	WT intein with G-1A, H73A mutation
MBP-WT(G-1A, H73A, N154A, S+1A)-Trx	This work	pKF205	pMST	WT intein with G-1A, H73A, N154A, S+1A mutation

MBP-M86(G-1A)-Trx	This work	pKF191	pMST	M86 intein with G-1A mutation
MBP-M86(G-1A, H73A)-Trx	This work	pKF192	pMST	M86 intein with G-1A, H73A mutation
MBP-M86(G-1A, H73A, N154A, S+1A)-Trx	This work	pKF207	pMST	M86 intein with G-1A, H73A, N154A, S+1A mutation
MBP-WT(G-1T)-Trx	This work	pKF213	pMST	WT intein with G-1T mutation
MBP-WT(G-1T, H73A)-Trx	This work	pKF215	pMST	WT intein with G-1T, H73A mutation
MBP-WT(G-1T, H73A, N154A, S+1A)-Trx	This work	pKF194	pMST	WT intein with G-1T, H73A, N154A, S+1A mutation
MBP-M86(G-1T)-Trx	This work	pKF195	pMST	M86 intein with G-1T mutation
MBP-M86(G-1T, H73A)-Trx	This work	pKF208	pMST	M86 intein with G-1T, H73A mutation
MBP-M86(G-1T, H73A, N154A, S+1A)-Trx	This work	pKF196	pMST	M86 intein with G-1T, H73A, N154A, S+1A mutation
MBP-WT(G-1L)-Trx	This work	pKF197	pMST	WT intein with G-1L mutation
MBP-WT(G-1L, H73A)-Trx	This work	pKF198	pMST	WT intein with G-1L, H73A mutation
MBP-WT(G-1L, H73A, N154A, S+1A)-Trx	This work	pKF199	pMST	WT intein with G-1L, H73A, N154A, S+1A mutation
MBP-M86(G-1L)-Trx	This work	pKF200	pMST	M86 intein with G-1L mutation
MBP-M86(G-1L, H73A)-Trx	This work	pKF201	pMST	M86 intein with G-1L, H73A mutation
MBP-M86(G-1L, H73A, N154A, S+1A)-Trx	This work	pKF203	pMST	M86 intein with G-1L, H73A, N154A, S+1A mutation
MBP-WT(G-1H)-Trx	This work	pKF55	pMST	WT intein with G-1H mutation
MBP-WT(G-1H, H73A)-Trx	This work	pKF56	pMST	WT intein with G-1H, H73A mutation
MBP-WT(G-1H, H73A, N154A, S+1A)-Trx	This work	pKF58	pMST	WT intein with G-1H, H73A, N154A, S+1A mutation
MBP-M86(G-1H)-Trx	This work	pKF63	pMST	M86 intein with G-1H mutation
MBP-M86(G-1H, H73A)-Trx	This work	pKF64	pMST	M86 intein with G-1H, H73A mutation
MBP-M86(G-1H, H73A, N154A, S+1A)-Trx	This work	pKF66	pMST	M86 intein with G-1H, H73A, N154A, S+1A mutation
MBP-WT(G-1F)-Trx	This work	pKF59	pMST	WT intein with G-1F mutation
MBP-WT(G-1F, H73A)-Trx	This work	pKF60	pMST	WT intein with G-1F, H73A mutation
MBP-WT(G-1F, H73A, N154A, S+1A)-Trx	This work	pKF62	pMST	WT intein with G-1F, H73A, N154A, S+1A mutation
MBP-M86(G-1F)-Trx	This work	pKF67	pMST	M86 intein with G-1F mutation
MBP-M86(G-1F, H73A)-Trx	This work	pKF68	pMST	M86 intein with G-1F, H73A mutation
MBP-M86(G-1F, H73A, N154A, S+1A)-Trx	This work	pKF70	pMST	M86 intein with G-1F, H73A, N154A, S+1A mutation

Table S5. ESI-MS-analysis of Int^N-peptides

	**$[M+H]^+$ [Da]**	**$[M+2H]^{2+}$ [Da]**		**$[M+3H]^{3+}$ [Da]**	
	**calc.**	**calc.**	**obs.**	**calc.**	**obs.**
**pep1**	1967.1	984.1	983.6	656.4	656.2
**pep2**	1981.2	991.1	990.6	661.1	660.8
**pep3**	1995.2	998.1	997.7	665.7	665.5
**pep4**	2011.2	1006.1	1005.6	671.1	670.8
**pep5**	2023.2	1012.1	1011.6	675.1	674.9
**pep6**	2047.2	1024.1	1023.6	683.1	682.8
**pep7**	2057.3	1029.1	1028.7	686.4	686.2
**pep8**	2023.2	1012.1	1011.6	675.1	674.8
**pep9**	2057.3	1029.1	1028.7	686.4	686.2

Supporting Figures

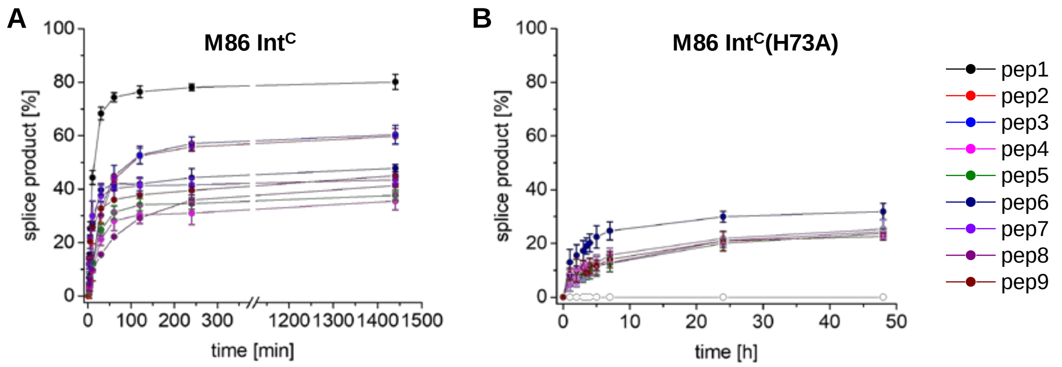

Figure S1. Time-courses of semi-synthetic protein *trans*-splicing. Reaction schemes are as shown in Figure 1A. Shown are the time-dependent analyses of the reactions as described in Figures 1 and 4. Aliquots were removed from the reaction mixtures at the indicated time points and analyzed. Yields of protein *trans*-splicing were determined by densitometric analysis of Coomassie-stained SDS-PAGE gels. Error-bars indicate standard deviations.

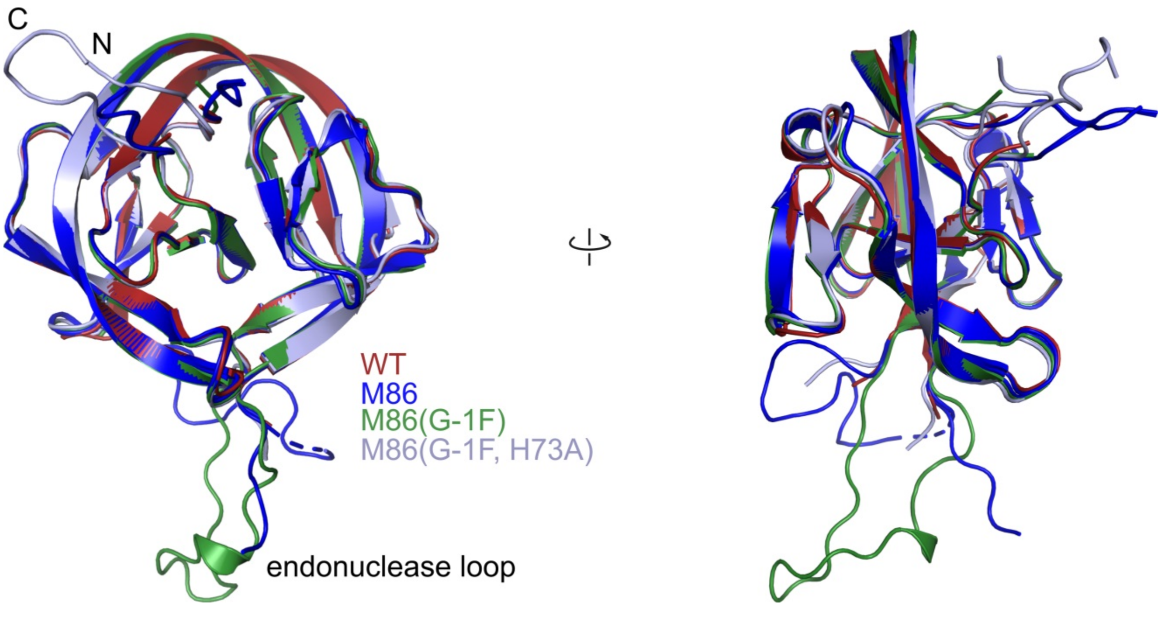

Figure S**2: Illustration of endonuclease loop and extein sequences.** Shown are two perpendicular views of an overlay of WT *Ssp* DnaB (PDB entry 1MI8; red), the two copies of M86 contained in the asymmetric unit of the crystal form investigated in this study (blue), M86(G-1F) (green) and M86(G-1F, H73) (light blue). Note the different orientations of the endonuclease loop, which is a consequence of different crystal environments.

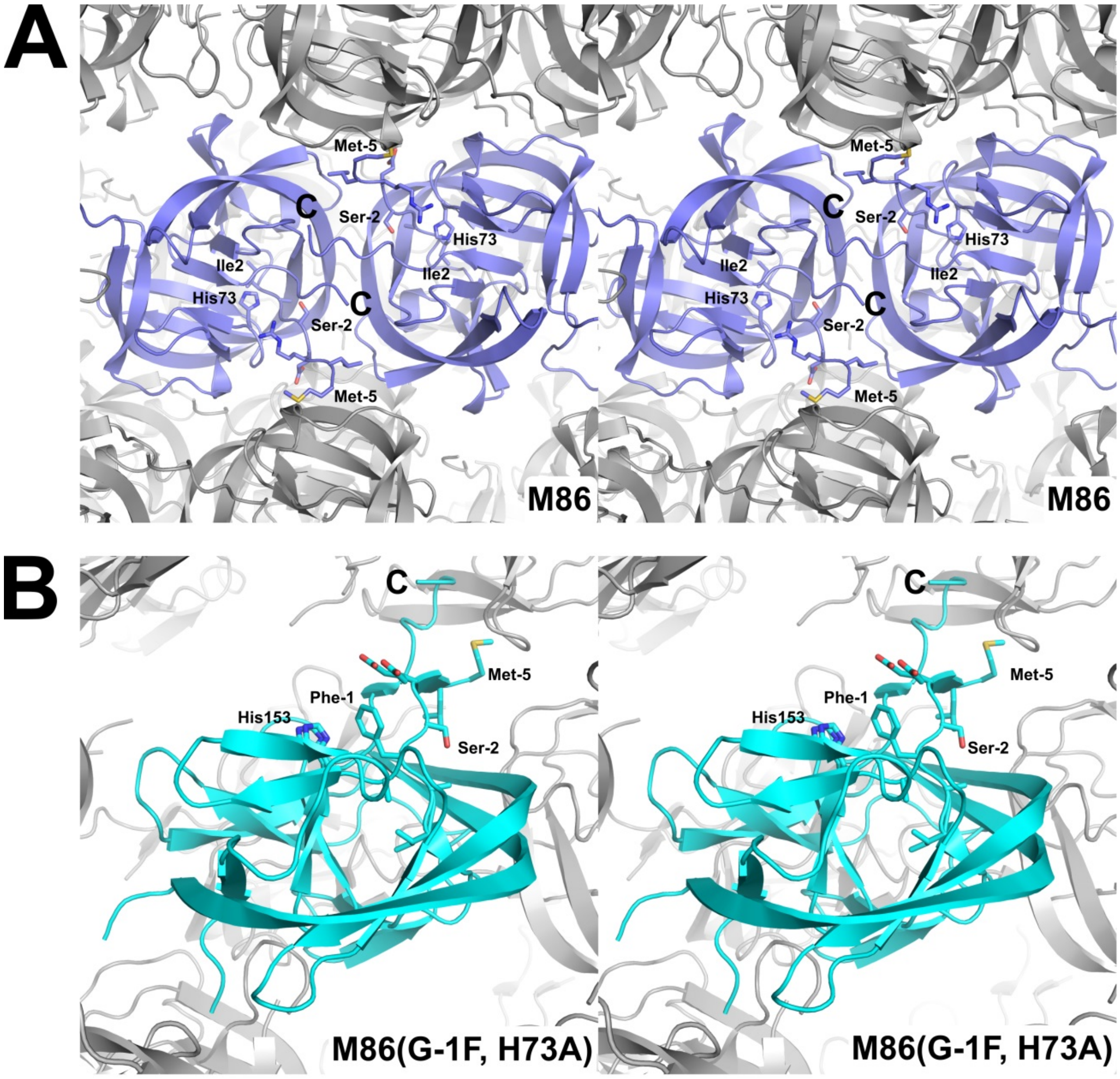

Figure S3. Cross-eyed stereo plots of the crystal packing environments of the N- and C-terminal regions. (A) M86 (blue) and (B) M86(G-1F, H73A) (cyan). The termini of WT *Ssp* DnaB (PDB entry 1MI8) and of M86(G-1F) are not involved in crystal contacts (not shown).

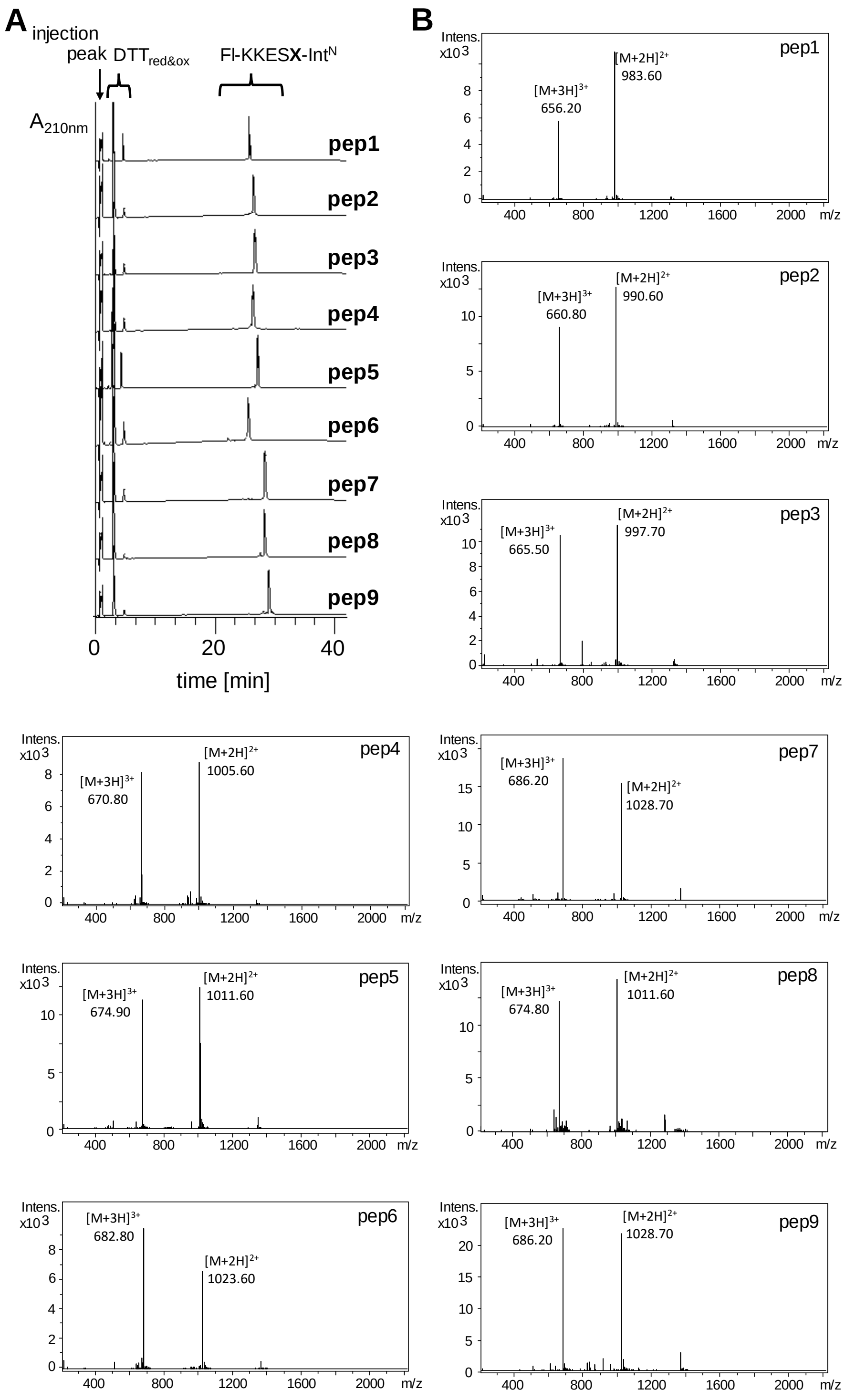

Figure S4. Purity of Int^N-peptides. (**A**) Shown are analytical HPLC traces. Note that the peptides appear as a double peak due to the 5,6-isomers of the carboxyfluoresceine moiety. (**B**) ESI-MS analysis. See Table S5 for an overview of observed and calculated masses. The contamination in **pep3** has a mass of 1589 Da (observed).

Supporting References

1. P. R. Evans and G. N. Murshudov, *Acta Crystallogr D Biol Crystallogr*, 2013, **69**, 1204-1214.
2. P. A. Karplus and K. Diederichs, *Science*, 2012, **336**, 1030-1033.
3. M. S. Weiss, *J. Appl. Cryst.*, 2001, **34**, 130-135.
4. V. B. Chen, W. B. Arendall, 3rd, J. J. Headd, D. A. Keedy, R. M. Immormino, G. J. Kapral, L. W. Murray, J. S. Richardson and D. C. Richardson, *Acta Crystallogr D Biol Crystallogr*, 2010, **66**, 12-21.
5. M. A. Johnson, M. W. Southworth, T. Herrmann, L. Brace, F. B. Perler and K. Wuthrich, *Protein Sci*, 2007, **16**, 1316-1328.
6. T. Klabunde, S. Sharma, A. Telenti, W. R. Jacobs, Jr. and J. C. Sacchettini, *Nat Struct Biol*, 1998, **5**, 31-36.
7. A. S. Aranko, J. S. Oeemig, D. Zhou, T. Kajander, A. Wlodawer and H. Iwai, *Mol Biosyst*, 2014, **10**, 1023-1034.
8. J. S. Oeemig, A. S. Aranko, J. Djupsjobacka, K. Heinamaki and H. Iwai, *FEBS Lett*, 2009, **583**, 1451-1456.
9. A. S. Aranko, J. S. Oeemig, T. Kajander and H. Iwai, *Nat Chem Biol*, 2013, **9**, 616-622.
10. Q. Wu, Z. Gao, Y. Wei, G. Ma, Y. Zheng, Y. Dong and Y. Liu, *Biochem J*, 2014, **461**, 247-255.
11. J. S. Oeemig, D. Zhou, T. Kajander, A. Wlodawer and H. Iwai, *J Mol Biol*, 2012, **421**, 85-99.
12. B. W. Poland, M. Q. Xu and F. A. Quiocho, *J Biol Chem*, 2000, **275**, 16408-16413.
13. R. Mizutani, S. Nogami, M. Kawasaki, Y. Ohya, Y. Anraku and Y. Satow, *J Mol Biol*, 2002, **316**, 919-929.
14. E. Werner, W. Wende, A. Pingoud and U. Heinemann, *Nucleic Acids Res*, 2002, **30**, 3962-3971.
15. Y. Ding, M. Q. Xu, I. Ghosh, X. Chen, S. Ferrandon, G. Lesage and Z. Rao, *J Biol Chem*, 2003, **278**, 39133-39142.
16. P. Sun, S. Ye, S. Ferrandon, T. C. Evans, M. Q. Xu and Z. Rao, *J Mol Biol*, 2005, **353**, 1093-1105.
17. B. P. Callahan, N. I. Topilina, M. J. Stanger, P. Van Roey and M. Belfort, *Nat Struct Mol Biol*, 2011, **18**, 630-633.
18. A. K. Dearden, B. Callahan, P. V. Roey, Z. Li, U. Kumar, M. Belfort and S. K. Nayak, *Protein Sci*, 2013, **22**, 557-563.
19. L. M. Kick, S. Harteis, M. F. Koch and S. Schneider, *Chembiochem*, 2017, **18**, 2242-2246.
20. C. Ludwig, M. Pfeiff, U. Linne and H. D. Mootz, *Angew Chem Int Ed Engl*, 2006, **45**, 5218-5221.
21. J. Binschik and H. D. Mootz, *Angew Chem Int Ed Engl*, 2013, **52**, 4260-4264.
22. J. H. Appleby-Tagoe, I. V. Thiel, Y. Wang, Y. Wang, H. D. Mootz and X. Q. Liu, *J Biol Chem*, 2011, **286**, 34440-34447.
23. H. Wu, M. Q. Xu and X. Q. Liu, *Biochim Biophys Acta*, 1998, **1387**, 422-432.
24. J. K. Bocker, K. Friedel, J. C. Matern, A. L. Bachmann and H. D. Mootz, *Angew Chem Int Ed Engl*, 2015, **54**, 2116-2120.
